# An Overview of Soil and Soilless Cultivation Techniques—Chances, Challenges and the Neglected Question of Sustainability

**DOI:** 10.3390/plants11091153

**Published:** 2022-04-24

**Authors:** Andre Fussy, Jutta Papenbrock

**Affiliations:** Institute of Botany, Leibniz University Hannover, Herrenhäuserstr. 2, D-30419 Hannover, Germany

**Keywords:** aquaponics, hydroponics, nutrients, sustainability, vertical farming

## Abstract

Resources such as fertile soil and clean water are already limited in many parts of the world. Additionally, the conventional use of arable land is becoming increasingly difficult, which is further exacerbated by climate change. Soilless cultivation systems do not only offer the opportunity to save water and cultivate without soil but also the chance to open up urban areas such as residential rooftops for food production in close proximity to consumers. In this review, applications of soilless farming systems are identified and compared to conventional agriculture. Furthermore, aspects of economic viability, sustainability and current developments are investigated. An insight into the most important soilless farming systems—hydroponics, aquaponics and vertical farming—is provided. The systems are then differentiated from each other and, as far as possible, evaluated in terms of their environmental impact and compared with conventional cultivation methods. Comparing published data analyzing the yield of hydroponic cultivation systems in comparison to soil-based cultivation methods enables a basic overview of the profitability of both methods and, thus, lays the foundation for future research and practical applications. The most important inert substrates for hydroponic applications are presented, and their degree of sustainability is compared in order to emphasize environmental impacts and affect substrate selections of future projects. Based on an assessment of the most important soilless cultivation systems, the challenges and developments of current techniques are highlighted and discussed.

## 1. Introduction

Biocapacity describes the ability of our planet’s ecosystems to regenerate. This capacity can be defined as the basic currency of all living systems on Earth. The ecological footprint measures both the available biocapacity and the demand that humans determine through all their activities [1,2,3,4,5,6,7]. Advances in technology and land management have increased global biocapacity by about 28% over the last 60 years [8,9]. However, this may be overestimated because the UN statistics did not include losses such as soil erosion, groundwater depletion and deforestation. In any case, this increase has not kept pace with the growth in overall consumption; Humanity’s Ecological Footprint, also estimated using UN statistics, has increased by about 173% over the same period [3,7,8,9] and now exceeds the planet’s biocapacity by 56%. This means that human activity is currently 1.56 times more than what the Earth can regenerate [9]. As with the 2008 economic crash, COVID-19 has also reduced human demand by almost 10% since 2020 [9]. It is unlikely that this will last, as the effect is not due to structural changes. In some circumstances, the global pandemic may even delay efforts to combat climate change and biodiversity loss [10,11]. Climate change in particular exacerbates the problem in that, for example, natural disasters and rising sea levels increasingly endanger aquatic products [12,13,14,15]. In addition, water and soil losses, desertification and salinization threaten our agricultural crops [16].

Soil is the most available growth medium (GM) for plants. It provides anchorage, nutrients, air, water, etc. for successful plant growth. However, different soil types sometimes also pose serious constraints to plant growth. Inappropriate soil pH, unfavorable soil compaction, poor drainage, degradation due to erosion, presence of pathogenic organisms and nematodes, etc. are some of them. Moreover, the conventional cultivation of crops, as open-field cultivation, requires resources such as space, (often) irrigation and (large) technical equipment and is weather-dependent. In some places, such as in or close to big cities, space for growing crops is limited, or in some areas, there is little fertile arable land due to unfavorable geographical or topographical conditions.

Conventional farming practices mainly involve such soil-bound methods and can cause a variety of negative effects on the environment. “Conventional” has historically been defined as the practice of growing crops in the ground, outdoors, often with irrigation and the active application of nutrients. The negative impacts of conventional agriculture relate not only to the growth conditions of the crops but in particular to the impact on natural ecosystems, including high and inefficient water demand, large land requirements, fertilizer use, soil degradation and loss of biodiversity [17,18]. With rising world hunger, the increase in food production has to keep pace. In particular, the rational use of water and land resources justifies the development of technologies that achieve optimized crop yields. Within greenhouse systems, the knowledge of plant water and nutrient uptake is crucial for the development of sustainable strategies that optimally support plant growth [19]. Compared to some alternative strategies, conventional agriculture consumes large amounts of irrigation with fresh water and fertilizers, with comparably low yields [9]. Hydroponics, aeroponics and aquaponics are agricultural systems that combine nutrient solutions, sometimes with inert substrates, instead of using soil for plant nutrition [20]. The need to develop fertile soils to obtain new sites for food production can be reduced with these approaches. Equally advantageous is the possibility of further increasing the yield per unit area with vertical farming approaches [21]. Furthermore, in a controlled environment, continuous production can be implemented throughout the year [22].

Protected cropping is defined as growing within, under or sheltered by structures such as cover material, shade cloths, plastic tunnels or greenhouses. These structures or materials help to optimize conditions for plant growth by being able to protect plants from pests or adverse weather conditions. The level of protection and control can range from an inexpensive canopy (e.g., fabric/cloth) in a field to greenhouses or complete controlled environment horticulture (CEH) systems [23].

Soilless plant cultivation in an almost completely controlled environment is a relatively modern cultivation technology and is used almost exclusively in greenhouses. Over a long period of time, attempts have been made to eliminate problems associated with the soil in greenhouses. These include soil-borne diseases as well as low soil fertility or high salt levels, as mentioned before. In particular, the development of suitable growing media with optimal physical and chemical properties over the past 30–40 years has led to hydroponic cultivation in greenhouses taking a leading role within cultivation technologies. Advances in plant nutrition and irrigation through modern fertilization approaches and automation technologies also have favored this development [24].

When soilless agricultural production systems are mentioned, it is mainly referred to as the techniques of “hydroponics”. The term hydroponics was derived from the Greek words *hydro*, meaning water, and *ponos*, meaning work. It is a method of growing plants using mineral nutrient solutions without soil [25]. Terrestrial plants can be grown only in the mineral nutrient solution or in an inert medium such as perlite, gravel or mineral wool. Hydroponics is, therefore, the technique of growing plants in soilless conditions with inert substrates or with their roots immersed in a nutrient solution without the use of any aggregate [26].

This article presents different hydroponic and related approaches and compares them with each other with regard to conventional, soil-based systems. One of the novelties of this article is the evaluation of a large amount of up-to-date available scientific data displaying the yield of soilless cultivation methods. This provides a preliminary assessment of the profitability of hydroponic cultivation of many globally used plant species compared to soil-based cultivation, which should support future research and practical applications. Such an assessment has not been performed so far. Likewise, the most important inert substrates for hydroponic applications are presented and compared in terms of their sustainability. Based on a comparison of the most important soilless cultivation systems, which also takes aspects of sustainability into account, the challenges and developments of current techniques are highlighted and put into context with the current scientific approaches. This way, an overview of new soilless cultivation systems is created, combining aspects of sustainability and economic viability. At the same time, it becomes apparent that sustainability, especially with regard to energy balancing, is a field of research that has hardly been investigated so far. The discrepancy between expectations regarding technologies and the high energy costs, mostly generated by fossil fuels, further underlines this problem.

## 2. Classification of Soilless Systems

The term soilless culture originally referred only to nutrient solution cultures without a support medium like soil. It now also includes growing plants with a nutrient solution with solid support media for anchoring. This technique is called a solid media, substrate culture or aggregate system. Hydroponic systems are further categorized into open (i.e., once the nutrient solution has been delivered to the plant roots, it is not reused) or closed (i.e., excess solution is recovered, replenished and recycled). In closed systems, the use of growing media is usually refrained from as they can complicate the recirculation process. Such systems that use growing media (container plants) can still be both closed and open. The closer classification is according to the techniques for distributing the nutrient solution to the plant roots [27,28].

By definition, hydroponics are soilless culture systems with inert substrates or without any aggregates, with plants whose roots reach into an aerated nutrient solution, which can be either flowing or static. Within the liquid or solution culture techniques represented in Table A1, two basic principles can be distinguished from each other. The first principle can be considered a circulating method (closed system) or continuous-flow solution culture, including, for example, the nutrient film technique (NFT) and the deep flow technique (DFT). Flow solution culture systems such as the DFT can provide a constant nutrient environment for roots. They are very well suited to automatic control but are subject to the rapid dehydration of plants if the flow of the solution stops for any reason. The second hydroponic principle can be called a non-circulating method (open systems) or static solution culture and can be further divided into three system techniques: the root dipping technique, the floating technique and the capillary action technique [27]. The main advantage of closed hydroponic systems compared to open systems is their water efficiency and nutrient consumption [28].

The techniques of solid media culture can again be divided into the hanging bag technique, the grow bag technique, the trench or trough technique and the pot technique, which are shown in Table A2. These techniques assume solid substrates. The selected medium must be flexible, friable, capable of retaining water and air and easy to drain. It must also be free of toxic substances, pests, pathogenic microorganisms and nematodes [27].

Substrate-based hydroponic systems dominate over water culture systems because the water-holding capacity of the substrate can provide a safety reserve in case, for example, a pump fails. In addition, the most common substrates provide better root aeration than water culture systems due to their porous nature, with the exception of aeroponics [29]. The containers for substrates can be bags, pots, other types of containers or troughs, and they determine the structure of the system [30]. Although the cost of the substrate is higher when it is packed in bags, bag culture is the most widely used type of cultivation on substrates. This is also because the bags can be standardized and are easy to handle, reducing labor costs and the risk of errors during installation. The bags are made of UV-resistant polyethylene film, white on the outside to reflect radiation and thus, avoid possible overheating, and black on the inside to prevent algae development [31].

## 3. Soilless Cultivation

### 3.1. Suitable Plant Species for Soilless Cultivation Applications

The basic requirement for the high productivity of crops is a suitable environment. A suitable environment for improved crop production is sheltered cultivation or hydroponic greenhouse cultivation. It is possible to grow cereals, vegetables, fruits, fodder crops, flowers, spices and medicinal plants in hydroponic greenhouses [32]. An overview of the crops that can be grown in hydroponic greenhouses and the state of research to date on the question of whether a crop-specific increase in yield can be expected compared to conventional agriculture is given in Table A3. The difference in yield sometimes was unexpectedly large and was due to the controlled environment, in the case of hydroponic greenhouse cultivation, as well as to the reuse of the nutrient solution. Overall, the quality of the hydroponic products, as well as their taste and nutrient composition, often were better than those in conventional soil-based cultivation. Various experimental findings show that leafy vegetables (lettuce, spinach, parsley, celery and Atriplex, etc.) can be grown comparatively easily and successfully using hydroponic systems [33]. So far, various vegetables have been grown in hydroponic systems. Examples are lettuce and chives in Trinidad [34], blueberries in Taiwan [35] and several crops in the greater Gaborone area in Botswana [36]. In countries with limited drinking water supplies, such as the Gulf States, the water-saving practice of hydroponics has been used to successfully grow various vegetables [37]. Lettuce and spinach are promising species for cultivation in integrated hydroponic and aquaculture systems. This is due to their fast growth and high nutrient uptake capacity. In hydroponic systems, the life cycle of lettuce can be significantly shortened compared to traditional growing methods. The NFT is particularly suitable for the cultivation of lettuce and allows for more than eight harvests per year. Horizontal and vertical hydroponic approaches have also been successfully tested for the yield optimization of lettuce crops [38]. There are significant differences in the productivity and nutrient contents of lettuce between soilless culture and conventional soil culture. Interestingly, the yield of soil-grown lettuce was actually higher in this case, and the nitrate content in the leaves was correspondingly higher, too [39]. Spinach has also been extensively tested in hydroponic systems in recent times. Spinach yields in a hydroponic system with peat moss compared to an aquaponic system with perlite and a traditional system led to similar results [40]. It was observed that the yield of aquaponically cultivated spinach was slightly higher than that of hydroponically cultivated spinach. Additionally, high mineral contents were found in hydroponically cultivated chard, lettuce and sweet basil as well as a high root/shoot ratio with a low nitrate content compared to plant material derived from conventional culture conditions [41]. However, in some observations, nutrient uptake and yield were lower in the hydroponic cultures.

Plants like tomatoes and peppers grow tall, and some platforms cannot hold them because of their weight, whereas drip systems provide enough stability. Nevertheless, various hydroponic systems are available for growing tomatoes, with the NFT being the most commonly used system. Regarding different substrates for hydroponically grown peppers, peat, in combination with perlite, might have the greatest positive influence on the growth characteristics and yield [42].

Cucumbers are also best grown with drip system irrigation. Celery, on the other hand, has shallow roots and responds well to ebb and flow systems. Radish is suitable for water culture. For fruit crops such as strawberries, blueberries and melons, the NFT provides an optimal environment. It meets the special requirements of the plants, e.g., regarding moisture. For melons, the ebb and flow system can also be used. Herbs such as chives are susceptible to drought stress and can, therefore, be cultivated well with the NFT [43]. Aside from vegetable products, strawberries and various cut flowers are, nowadays, grown commercially in diverse hydroponic systems [33]. Additionally, hydroponic systems can be used to produce very clean, high-quality herb and root crops [44]. The yield of basil seems to be affected more by cultivar selection than by the choice of a hydroponic production system. Therefore, hydroponic basil producers should select basil cultivars based on flavor and yield, while hydroponic systems should be selected based on operational preferences [45].

### 3.2. Inert Substrates for Soilless Cultivation

#### 3.2.1. General Aspects

Substrates or growth media (GM) include materials that, alone or in mixtures, can ensure better plant growth conditions than agricultural soil in at least one aspect [46]. So far, substrates could be used for the production of high-quality vegetables and ornamentals as well as for plant propagation. Table A4 gives an overview of substrates that can be used hydroponically. Generally, a mixture of substrate components and additives is used in the horticultural industry. These additives include fertilizers, liming agents and biological control or wetting agents. Substrate components, again, include combinations of different substances. These can be organic as well as inorganic [46]. In the commercial hydroponic production of vegetables and cut flowers, substrates such as rockwool, perlite or coconut coir are used. All inorganic substrates come from natural sources and are partly industrially pre-processed. Rockwool is suitable due to its light weight and easy handling. Until 20 years ago, mainly rockwool sheets were used as hydroponic substrates. Since then, a lot of knowledge has been gained about the use and comparison of different substrates. Perlite was found to be the most suitable substrate for hydroponic use in cucumber cultivation [47]. Recycled polyurethane-ether foam was also suitable for increasing yields, although it also increased water consumption. Perlite quickly became a well-established substrate throughout Europe for hydroponic applications and is used, in particular, due to its low cost [48]. Gravel and sand have been used in older production systems but are less efficient due to their porosity. In addition to rockwool and perlite, various inorganic GM, such as pumice, zeolite, tuff, volcanic porous rock, expanded clay granules and vermiculite have been used in substrate cultures [49]. Organic substrates can be synthetic, such as polyurethane, or consist of natural organic substances, such as peat or wood-based substrates. Peat [50], composts [51], bark [52], wood residues [53] and coconut coir [54] are the most commonly used. Peat is still the most popular substrate component in horticulture. Peat is mainly used in tree nurseries and ornamental plant farms, while other substrates are preferred for vegetable and cut flower crops. Peat is popular due to its relatively low cost and excellent chemical, biological and physical properties, with low nutrient content, low pH, high water-holding capacity, high air space and low weight [49]. The current trend towards the use of biochar and hydrochar, a solid product formed during hydrothermal carbonization, is interesting to observe. Biochar has advantageous properties of low bulk density, high cation exchange capacity (CEC) and high nutrient-holding capacity. Thus, it reduces nutrient leaching [55]. On the other hand, the properties of biochar are not homogeneous, while the pH value and the production costs are relatively high. It can be seen that both inorganic and organic materials have their advantages and disadvantages as substrate components.

#### 3.2.2. Chemical Properties

When assessing the chemical properties of growing media, the most important criteria are pH, CEC and nutrient concentrations [46,49]. For most plant species, the pH in the root environment for optimal nutrient availability is between 5.5 and 6.0. In general, a lower pH and lower nutrient and salt concentrations are preferable for crop production.

#### 3.2.3. Biological Properties

A substrate needs to be free of pests, pathogens and weeds and be biologically stable and non-toxic. The use of forestry products as well as immature compost is partly problematic with regard to the associated phytotoxicity. For example, high potassium and manganese content [52] or the presence of phenolic compounds [56], terpenes, organic acids and fatty acids [57] can cause such toxicity problems [58]. Methods such as composting, aging, leaching, washing and mixing have been used to reduce or eliminate phytotoxic substrate properties [46,56,59]. Furthermore, different structures and stabilities can be attributed to mineral and organic substrates, which in turn, influence the functionality of the bacterial community [60].

#### 3.2.4. Environmental Perspective

The increasing environmental awareness of consumers, the continuous destruction of ecologically important peatlands and a pervasive waste problem have forced the horticultural industry to initiate changes in its practices. In particular, the search for alternatives to the limited availability of peat has been driven in recent years [46,54]. This has included concerns about removing peat from peatland ecosystems, which are unique habitats for wildlife and equally important for water quality and the hydrological cycle as well as carbon sequestration. As a result, the use of peatlands has been reconsidered towards less exploitation, and extensive research has been conducted [49]. There are also difficulties with other substrates such as mineral wool, including the existing disposal problems and the high energy consumption during production. The international trend in GM development relies on the use of local, natural resources and renewable raw materials. Especially in industrialized countries, the reuse of waste has become highly desirable [61].

Nowadays, coconut fiber as a substrate has a high value in open cultivation. The waste product from the husk of coconut fruits is light and has good aeration and water retention properties. Despite this, the long transport distances from the cultivation areas in Indonesia, Sri Lanka, India, the Philippines and Latin America can be criticized [54]. Nevertheless, coconut fiber is now the most popular substrate in many areas, ahead of rockwool [62].

Perlite is the most impactful substrate, as highlighted by its life cycle assessment (LCA), followed by rockwool and vermiculite [63]. The most sustainable ones, instead, are sand and bark. Sand has a low carbon footprint. In addition, bark, in the total impact analysis, seems to be highly sustainable. For perlite, the two results are in disagreement: it has a high total impact, but a very low carbon footprint compared to the other substrates. In another life cycle costing analysis, it appears that peat is the most expensive substrate, while sand is the cheapest one. In addition, the use of peat substitutes, such as compost or biochar, can significantly reduce the carbon footprint in hydroponic systems [64,65].

#### 3.2.5. Choice of Growing Medium

The choice of material to be used as substrate depends on the plant species to be grown. Therefore, the properties of growing media have to meet the requirements of plant production, which in turn, are determined by plant biology and the cultivation method. Of course, costs also play an important role. However, the development currently goes beyond the production-driven decision criteria. In order to generate sustainable future prospects, substrates should also be environmentally friendly and consumer-oriented [54]. Overall, LCA is coming to the fore for the classification of substrate ingredients, based on their environmental impact, sustainability, environmental protection aspects and the use of “green technologies” for their production. Judging by this, sand is the most sustainable substrate for hydroponic cultures, followed by bark. Biochar can also minimize the negative effects on our environment caused by the use of substrates and, additionally, brings physically advantageous properties.

### 3.3. Plant Population Management in Hydroponics

#### 3.3.1. Control of Pathogens

The application of pesticides is generally reduced in hydroponic systems. With fewer pest problems and the constant feeding of nutrients to the roots, productivity in hydroponics is high, despite the limited plant growth that could occur due to low levels of carbon dioxide in the atmosphere or limited light supply in closed and sub-optimally ventilated environments. To increase yield further, some greenhouses inject carbon dioxide into their environment to help growth (CO_2_ enrichment), prolong the light period or increase the light supply to control vegetative growth, etc. [32].

Maintaining a non-pathogenic environment in the root zone is critical for good plant vigor under soilless crops. It is extremely difficult to achieve this and crucial to minimize the population of plant pathogens in the root zone [66]. A common disease in hydroponic solutions is wilt caused by *Fusarium* spp. and *Verticillium* spp. Species of *Pythium* spp. and *Phytophthora* spp. destroy everything except the main roots. There are no effective fungicides that can be safely used in hydroponics [67]. Only metalaxyl has been shown to be highly effective in controlling *Pythium* spp. on vegetable crops, but it is not registered for use. In addition to environmental regulations that restrict the discharge of pesticides into the environment, there are no appropriate agents against many diseases, and often disease-causing pathogens develop resistance to pesticides over time [68].

In general, non-chemical methods can be distinguished from chemical methods for the disinfection of nutrient solutions. In the case of non-chemical methods, the nutrient solution is not chemically changed during disinfection so that no residues can be found afterwards. Non-chemical methods include heat treatment, filtration, and the use of UV radiation. Heat treatment of the nutrient solution has been shown to be effective in keeping the root zone free of pathogens [66]. Root death of tomato caused by *Pythium* spp. was overcome by heating the nutrient solution up to 20–22 °C. In aeroponic systems with heated nutrient solution, the roots of ginger plants matured even faster and produced slightly higher fresh rhizome yields than plants in the same medium without bottom heating [32]. Different species are neutralized by different temperatures in this process. In general, a temperature set point of 95 °C and an exposure time of at least 10 s are sufficient to kill most undesirable organisms. It should be borne in mind that energy can be saved with the aid of heat exchangers. Nevertheless, the origin of the energy is a determinant for the sustainability of this method [68].

Another method to maintain a non-pathogenic environment is filtering the nutrient solution. There are different types of filters depending on the particle size of the undissolved material. Fast sand filters, for example, are suitable for filtering out large particles. Smaller, synthetic filters are often used after nutrient enrichment to remove undissolved fertilizer salts or precipitates that might otherwise cause clogging. These filters are commonly also used prior to disinfection by heat treatment, UV irradiation or ozone treatment. With smaller pore sizes of the filters, the resistance for the aqueous solution also increases. In addition to the very small-pore filters suitable for this purpose, this also requires correspondingly high pressure. In addition, the filters must be cleaned frequently [68].

UV radiation with a wavelength range between 200 and 280 nm (UV-C) and an optimum at 254 nm can also be used to kill microorganisms by damaging their DNA. Recommended for the disinfection of organic components is treatment at 185 and 254 nm [69]. The use of UV radiation is also suitable for aquaponic systems and efficiently inactivates coliforms [70].

Moving on to chemical disinfection methods, ozone is generated from dry air and electricity using ozone generators according to the following formula:

3O_2_ ↔ 2O_3_. (1)

When the air is enriched with ozone, it is injected into the water to be disinfected and should be exposed for one hour. The method can, by now, be used to reliably neutralize viruses as well as all other pathogens without any safety problems [71]. Ozone treatment affects the microbial populations in plant cultures [72]. Humans should not come into contact with ozone, as even small amounts can cause irritation of the mucous membranes. Like UV rays, ozone treatment affects iron chelates. Consequently, the addition of iron must be increased and iron deposits in the system counteracted.

The use of hydrogen peroxide (H_2_O_2_) as a strong, unstable oxidant with the reactive formation of water and a free oxygen radical is now considered inefficient, even though it is inexpensive. It is still used to clean systems but has been replaced by other methods for active disinfection in running systems. To kill viruses, concentrations of about 0.05% are needed, which also damage plant roots [73].

Sodium hypochlorite (NaOCl) is commonly used as an inexpensive compound in swimming pools for water treatment. It reacts as follows:

NaOCl + H_2_O ↔ HOCl + NaOH (2)

In this process, the resulting compound of oxygen and chloride breaks down directly into its components in strong oxidation and reacts with all available organic substances. At high temperatures and with exposure to air, the effective substance decomposes more quickly, and phytotoxic NaClO_3_ is formed [74]. Furthermore, viruses cannot be rendered harmless efficiently with sodium hypochlorite treatment, and some bacterial pathogens also survive the treatment due to their spores [73]. Moreover, this method increases the concentrations of Na^+^ and Cl^-^ in closed systems, which makes it necessary to wash out the nutrient solution in order to avoid negatively affecting plant growth. Nevertheless, sodium hypochlorite is used and recommended for disinfection in commercial farms as a cost-effective and useful method.

For farmers, efficiency and cost play the biggest roles when choosing disinfection methods. High disinfection efficiencies are achieved by heat treatment, UV irradiation and ozone treatment. Ozone treatment is the most expensive method due to purchase and maintenance costs, whereas heat treatment and UV irradiation also cause high annual costs, but with lower initial investments. Especially farms larger than one hectare use heat treatment or UV irradiation. For smaller farms, the more cost-effective purchase of an inefficient and slower sand filtration system may be recommended. Sodium hypochlorite and hydrogen peroxide are not recommended due to their insufficient efficiency in killing pathogens [68]. As a biocide, the use of sodium hypochlorite is banned in the EU for such applications anyway. With all chemical methods, it should be borne in mind that they are not very selective in distinguishing between pathogens and other organic components, so non-chemical methods should be used for pre-treatment. The residues of chemical applications often react with biofilms in the system’s pipes, causing them to detach and clog the system.

#### 3.3.2. Physico-Chemical Parameters That Need to Be Controlled

The most important parameters for plant production in hydroponic systems are the pH, temperature and nutrient concentrations [75]. The pH can be altered by the addition of acid or alkaline solutions, resulting in either alkalization or acidification. Controlling the pH by adding salts is a complementary procedure. In practice, nitrogen is added in the form of ammonium and nitrate. If only ammonium was added, the pH would be lowered, and if nitrate was added alone, the pH would rise accordingly [76]. In addition to the pH value, temperature also plays a decisive role in the uptake of nutrients.

The oxygen content is also temperature-dependent, whereby at higher temperatures, there is generally less oxygen in the water and, at the same time, increased demand by the plants. Insufficient amounts of oxygen in the water lead to reduced root respiration and, thus, to lower nutrient and water uptake. In addition, root tissue may die and the plant may become more susceptible to pathogens [77]. The dissolved oxygen concentration should, therefore, be higher than 3 mg/L [78].

#### 3.3.3. Supply of Nutrients

In hydroponics, careful monitoring of the system is required due to the limited nutrient buffering capacity of the system and the risk of making rapid changes [32]. Two aspects of nutrition need to be considered. Firstly, the supply of nutrients by the nutrient delivery system, and secondly, the response of the plants to the nutrients must be considered. Critical levels for commonly used nutrients have been determined for many crops. The frequency and quantity of the nutrient solution administered depend on the type of substrate used (volume and physicochemical properties), the crop (species and stage of development), the size of the container, the cultivation and irrigation systems used and the prevailing climatic conditions. When a nutrient solution is supplied continuously, plants can take up ions at very low concentrations. It has been reported that a large proportion of the nutrients are not used by the plants, or at least their uptake has no effect on production. On the other hand, highly concentrated nutrient solutions lead to excessive nutrient uptake, so toxic effects can be expected. Parameters of the nutrient solution such as temperature, pH, electrical conductivity, oxygen content and others must be precisely adjusted to the needs of the respective plant species in order to create optimal growth conditions [79]. The solution should be directly available to the roots, although it is advisable to avoid wetting the leaves in order to reduce damage and the occurrence of diseases. Under no circumstances should the plants suffer from water stress, as this impairs growth [80]. To conserve resources, the excess nutrient solution drained from the containers during daily watering should be reused during the next watering. However, excess nutrient solutions should be monitored for the growth of algae and the development of other undesirable organisms and disinfected as necessary before reuse.

### 3.4. Assessment, Current Situation and Future Perspectives for Hydroponics

#### 3.4.1. Advantages of Hydroponics

There are many advantages of growing plants in soilless culture over soil-based culture [67]. Soilless culture offers opportunities to provide optimal conditions for plant growth and, therefore, higher yields that are consistently reliable can be obtained compared to open-field agriculture. Hydroponics produce the healthiest plants and cultivation is clean and relatively easy, requiring little effort. Nutrients are delivered directly to the roots, and as a result, plants grow faster with smaller roots, plants can be grown more densely and only 1/5 of the total area and 1/20 of the total water is needed to grow plants under soilless culture compared to soil-based culture [81]. Nevertheless, there still is a chance for some soil-borne pests, diseases or weed infestation to occur in the system, and the control of such diseases is absolutely necessary as they can spread rapidly. Overall, soilless culture provides efficient nutrient regulation and higher planting density and results in higher yield per hectare along with better quality produce. It is especially suitable for those regions of the world that lack arable land or fertile soil for agricultural culture [67].

#### 3.4.2. Disadvantages of Hydroponics

Despite the many advantages, soilless cultivation has some limitations. Its application on a commercial scale requires technical knowledge and high initial investment, although yields are high [82,83]. Considering the cost expenditure, soilless culture is limited to high-value crops. However, once the system is set up, it can be cheaper than running a conventional farm [28]. Plants in a hydroponic system are supplied via the same nutrient solution, which means that plants within the system should have the same requirements. In addition, waterborne diseases can easily spread from one plant to another [84]. Great care is required in controlling plant health. The dangers of direct consequences of incorrect fertilization have to be mentioned as well [85]. High temperatures and inadequate oxygen supply can limit production performance and cause crop failure. Unfortunately, hydroponic systems are fundamentally dependent on electricity and require expensive generators to compensate for power outages. Maintaining pH, electrical conductivity and the right concentration of nutrients in the medium is of utmost importance. Finally, light and energy supply are usually required to operate the system under a protected structure. The high energy inputs that are required to operate the system are a serious problem [29]. For this, aspects of sustainability have to be considered as long as a large part of our energy is generated from fossil fuels and with climate-damaging methods. A detailed LCA needs to be carefully done for a particular system, and the results need to be evaluated from a sustainability point of view.

#### 3.4.3. Global Hydroponics Market and Commercial Production

Historically, in the 1960s and 1970s, commercial hydroponic farms were developed in Abu Dhabi, Arizona, Belgium, California, Denmark, Germany, Netherlands, Iran, Italy, Japan, the Russian Federation and other countries. In the 1980s, many automated and computerized hydroponic farms were established around the world. Some hydroponic kits became popular in the 1990s [86].

The rapid gain in the popularity of soilless cropping systems around the world in the last three decades can be attributed to the disadvantages caused by cultivation in the soil (including soil-borne pathogens). Due to the extensive control and preventability of diseases, soilless cultivation systems are becoming increasingly important in protected cultivation. Simple greenhouse constructions to exploit favorable climatic conditions also play a supporting role. With soilless systems, even areas with saline, sodic or non-arable soils with poor structure, which make up a large part of the world’s agricultural land, can be used effectively for agriculture. High-quality and consumer-oriented agriculture with a focus on environmental and sustainability aspects will continue to influence the cultivation of our agricultural products in the near future. Nations that rely on large and modern greenhouses against less favorable climatic conditions need to make large investments to maximize yields and achieve consistently high product quality through complete control of all growing conditions. The installation of a hydroponic system is only a small part of the total cost and is a way to avoid the risk factors regarding soils. With simple greenhouse constructions, which only serve to improve the climatic conditions, e.g., to increase the temperature and to protect the plants from environmental influences, it only makes sense to establish a technically complex hydroponic system for a limited number of crops or in certain growing regions. Often, the initial costs for a complex, automated hydroponic system are too high as long as the soil does not lead to specific problems or the available space is limited. Hydroponic culture has been recommended when it could avoid critical soil culture problems, when water resources were limited or when the environmental impact of nutrient leaching was severe. This seems to be the main reason for the so-far lower spread of commercial soilless cultivation systems in countries characterized by a Mediterranean climate, for example [31].

In 2016, the global market for hydroponically grown products was estimated at USD 21,204.5 million. The global hydroponics market specifically includes tomato, cucurbits, lettuce and leafy vegetables, peppers and other food crops. Among these, tomato cultivation forms the largest market segment and accounted for 30.4% of the total hydroponic market in 2018 [33]. The hydroponic cultivation of tomato, lettuce and other leafy vegetables is predicted to grow in terms of their market share. This is also due to the attitude of consumers, whose awareness of quality products from greenhouses has been steadily increasing. Particularly in Europe and Asia, a trend towards production in greenhouses is recognizable. Europe has currently been the largest market for the use of advanced hydroponic techniques. The Asia-Pacific region has been the second-largest market for hydroponic products and is expected to grow steadily. The leading nations in hydroponic technology have been the Netherlands, Australia, France, England, Israel, Canada and the United States. With 13,000 ha under hydroponic cultivation for tomatoes, peppers, cucumbers and cut flowers, the Netherlands has been the world leader in the use of hydroponic technology [87]. The Netherlands has already produced 50% of the value of all fruits and vegetables grown in the country in hydroponic systems. Australia has also achieved a market value of about USD 300–400 million with hydroponic products such as vegetables, herbs and cut flowers, which corresponds approximately to 20% of the total value of vegetable and cut flower production [88]. Australia has also been the largest producer of hydroponically grown lettuce, globally. In addition to that, their hydroponic production of strawberries has exceeded the hydroponic production capacity of the United States. Furthermore, the hydroponic production of cut flowers in Australia has almost been as large as in the United States. Moreover, countries like Canada and Spain have expanded their acreage for commercial hydroponics. Japan has pioneered rice cultivation with the use of hydroponic technology [80].

Large quantities of berries, citrus fruits and bananas have been cultivated hydroponically in Israel’s dry and arid climate. Currently, it can be generalized that the demand for hydroponic cultivation has increased in all developing and developed countries. Hydroponic approaches are particularly suitable for alternative solutions such as the utilization of fallow land with poor soil quality but high water supply, for example, in India. Space reclamation for agricultural production in large cities such as Delhi, Chandigarh, Noida and Bangalore can hardly do without hydroponic techniques. Already today, city dwellers grow some leafy vegetables and small herbs and spices for fresh consumption on their rooftops and balconies [33].

### 3.5. Future Scope of Hydroponics

When talking about controlled environment agriculture, one of the greatest innovations of our time is computer technology. Technologies such as crop monitoring and mobile apps help farmers make decisions about “when”, “where”, “how” or “what” to plant, irrigate, fertilize and control. The benefits of these innovations include giving farmers greater control over production, increased and more sustainable yields, and greater flexibility to respond to changes in weather. In this context, sensor technologies, for example, enable the constant monitoring and identification of the crop as well as some self-controlled regulatory mechanisms. Automation will help agriculture to make production even more efficient and to control agricultural products at the plant level with less effort. With modern agricultural engineering, farming can be extended to new means, places and areas [28]. It would also be desirable for the consumer to save on pesticides in cultivation, which are used to disinfect the soil or to combat soil-borne pathogens, too.

The history of hydroponics goes back to before these innovations. As early as the 1970s, hydroponics was recognized as a promising technology and its massive spread was predicted. It is still true that hydroponics has been one of the fastest-growing sectors of our modern agriculture and may well dominate food production in the future [89]. As the population will continue to increase and cultivable land tends to decrease due to inefficient land management, people will turn to new technologies, such as hydroponics and aeroponics, to provide adequate ways of crop production [26]. To get an idea of the future of hydroponics, one has to take a look at the first applications of this once new science [32]. The land around Tokyo is extremely valuable due to the increasing population. To feed the people while conserving land, the country turned to hydroponic rice production [80]. Here, the rice is harvested in underground vaults without the use of soil. Thanks to environmental control, four harvest cycles can be carried out per year instead of the traditional one-year production [86].

Hydroponics has also been used successfully in Israel, for example. There, water is rather scarce due to the dry climate. A company called Organitech has grown plants in shipping containers about 12 m long using hydroponic systems. These are used to cultivate large quantities of berries, citrus fruits and bananas that could normally not be grown in Israel’s climate [29]. Hydroponic techniques produce a yield up to 1000 times greater than that produced annually on a comparably sized conventional area. One of the biggest advantages is that the process can be fully automated and controlled by robots that work in a similar way to assembly line production [86].

There has been much discussion in the scientific community about the potential of hydroponic systems in third world countries where water supplies are limited [26,32,80,89]. One obstacle is the high initial cost of setting up hydroponic systems, but in the long run, as with any technology, costs will come down, making the use of hydroponics much more realistic [32,66,80]. Hydroponics, thus, has the potential to feed millions of people in the future in areas of Africa and Asia where both water and crops are scarce, or to effectively use limited land in drastically developing large cities.

Hydroponics is, therefore, likely to be important for the future of space programs. In this regard, NASA is relying on extensive programs to further develop hydroponics, both for current space exploration and for future, long-term projects, such as colonizing Mars or the Moon. Since we have yet to find cultivable soil away from our planet that can support life in space, and the logistics of transporting soil on space shuttles seem impractical, hydroponics could play a supporting role in the future of space exploration [29]. The advantages of hydroponics in space are twofold: it offers the possibility of cultivating a greater variety of food crops and it can include a biological aspect called the bioregenerative life support system. This simply means that plants absorb carbon dioxide as they grow and provide new oxygen through the plant’s natural growth process. Obviously, this will be important for the long-term planning of both space stations and cities on other planets [32].

### 3.6. Preliminary Impact Assessment of Hydroponics

Greenhouses with hydroponic systems have been proposed as sustainable and controlled systems for growing food in urban areas. In this context, nutrient management in hydroponic agricultural systems has been an ecological challenge, the efficiency of which could be improved by the use of recycling. Therefore, three options were analyzed for nutrient recovery in hydroponics. Among them were direct leachate recycling (DLR), chemical precipitation (CP), and membrane filtration (MF), and their environmental performance was also investigated through LCA. The assessment showed that leachate recirculation (DLR) is the most environmentally friendly option in terms of global warming [90]. In contrast, CP and MF have three- and five-times greater impacts on our climate, respectively. Nevertheless, all three alternatives showed lower eutrophication potential compared to conventional agriculture. Meeting crop nutrient needs sustainably by reusing wastewater can save between 44 and 52% of global warming impacts with the DLR process or the MF process compared to conventional cultivation. Urban agriculture and its sustainability can be promoted through such circular principles.

In relation to this, a similar approach showed that hydroponic water reuse systems can be operated economically viable, that their products have a high level of product confidence and that their ecological effects can be positive if appropriate landscaping measures are taken [91]. Nevertheless, landscaping, as well as acceptance measures, should be carried out to accompany hydroponic crop production to improve its social and ecological impacts.

Other researchers have investigated the environmental impact of three hydroponic tomato production systems using LCA. Two NFT systems and one drip system with granulated rockwool were used for production. All inputs and outputs of each hydroponic system were divided into structural materials, cultivation inputs and waste. Structural materials and wastes accounted for a much smaller share of the environmental impacts. The environmental impact of fertilizers as a result of production was the highest. Water consumption, on the other hand, had a much lower environmental impact in all systems. However, all systems had different water consumption levels, with drip irrigation having the lowest water consumption. The impact of fertilizers could be minimized through a proper irrigation schedule and needs to be studied and improved in more detail as it probably has the most visible environmental impact [92].

As well as that, it was shown that the growing medium, the pots, the electricity demand, the transport of raw materials and the product deliveries have the greatest impact in ecological terms on hydroponic systems [93]. Paper pots can replace those made of plastic, resulting in large savings in greenhouse gas emissions and fossil resource consumption. Coconut fiber can replace conventional garden soil, also allowing for more environmentally friendly crop production. To further reduce the impact of hydroponic and related systems, resource-efficient steps are needed to improve the impact of high electricity demand, in particular. In addition, more symbiotic exchange relationships should be developed to harness urban waste and by-products.

## 4. Aquaponics

### 4.1. General Aspects of Aquaponics

Aquaponics is a sustainable food production system that combines hydroponics with aquaculture, in which the waste produced by farmed fish can be used as a nutrient source for plants. The nutrient-enriched water from the fish tank is used to feed the plants after being processed by nitrifying bacteria (*Nitrosomonas* spp. and *Nitrobacter* spp.), which convert ammonium as well as highly toxic ammonia into nitrate in two sequential chemical reactions. The nitrifying bacteria form biofilms in biofilters or in the substrate of the hydroponic system. In turn, excessively high oxygen content in the water can inhibit denitrification by bacteria [94].

The water cleaned by the plants is then returned to the fish [77,95]. This system combines efficient nutrient use with the minimization of water consumption, enabling production in areas with limited water supply. Only a small proportion of the total system’s water needs to be changed per day. Furthermore, the chemical fertilization of the plants might partly not be mandatory as the wastewater of aquaculture often has similar nutrient parameters as the nutrient solution of hydroponic systems. This does not necessarily apply to potassium, calcium and iron, so adding these nutrients makes sense for optimal plant growth [77]. Nitrogen and phosphorus, on the other hand, are abundantly available to the plants in the aquaponic system [96].

Aquaponics, thus, has both economic and environmental advantages. In addition to the efficient use of space, costs are reduced by the fact that high-cost water treatments for fish cultivation can be replaced by cheaper systems. In such a low maintenance cost alternative, the wastewater is first coarsely filtered and then treated in the biofilter by nitrifying bacteria. For the environment, the aspects of phytoremediation and the avoidance of the release of nutrients via wastewater are particularly advantageous and can additionally reduce maintenance costs.

In addition, pesticides are not allowed in fish farming, which makes it necessary to use biological techniques to protect plants and fish [77]. Despite the advantages, the aquaponic production system in Europe is still at an early stage of development, not least due to the fact that the bureaucratic requirements and regulations of the European Union have complicated the progress. In addition, aquaponic products cannot be sold as organic or subsidized in their production. Nevertheless, there are some relatively small companies producing and offering aquaponic products in the EU. The United States already has some medium-sized companies in the aquaponic sector. In addition, they established a certificate for organic aquaponic cultivation in 2008 [97].

As is well known, unused nutrients lead to eutrophication [13,98]. In conventional fish farming, up to two-thirds of the nutrients remain unused [99]. Nitrogen and phosphorus compounds in particular pollute the environment and lead to increased growth of phytoplankton and algae, including toxic algae [100,101]. This can result in anaerobic zones, high fish mortality and a loss of biodiversity [102,103]. In particular, an ammonium surplus and ammonia and nitrite from the degradation of proteins have a direct toxic effect on many aquatic organisms [100,104].

### 4.2. Advantages of Aquaponics

The benefits of aquaponics include high water-use efficiency, the use of a minimal amount of fertilizer, the saving of pesticides and antibiotics, the elimination of soil, the combination of crop and fish production and the reduction of the harmful input of aquacultural waste in the environment. Although data have still been thin in some areas, aquaponics already has a reputation as one of the most sustainable forms of agriculture [105,106]. The majority of aquaponic systems function as recirculating aquaculture systems (RASs), with plants uptaking nutrients from fish waste. No water is lost in the process except for splash water and due to the processes of evaporation, transpiration by plants and necessary withdrawals. RASs use 90–99% less water than conventional aquaculture systems [107].

Several studies have found that aquaponic systems typically consume between 0.3% and 5.0% of the total system’s water per day [108,109]. In comparison, some basic hydroponic recirculating systems require complete nutrient replacement every 2–3 weeks, making the water-use efficiency of hydroponic systems look comparatively poor [110,111]. In general, aquaponic systems use at least 50% of the nutrients originally provided by fish food as plant fertilizer. Again, there is a saving of a substantial amount of fertilizer compared to hydroponics, and the production of fertilizer accounts for a large proportion of the energy required for agriculture. Aquaculture farms require a filtration system to remove toxic compounds such as ammonia, nitrite and suspended solids from the system. These compounds, if not properly managed, can leach into neighboring environments and cause eutrophication of the water. Considering that aquaculture, as the fastest growing agricultural industry in the world, has been projected to provide 54% of the estimated 200 million tons of fish needed by 2030, it could possibly have an immense impact on the environment [112]. Due to its independence from land, the use of aquaponic systems can be optimized in controlled environments in urban areas. This can help mitigate potential land scarcity. Aquaponics has the potential to use the resources of our agriculture much more efficiently.

### 4.3. Disadvantages of Aquaponics

Achieving resource efficiency has been poorly demonstrated in previous trials. The challenges to establishing a successful and sustainable aquaponic operation are numerous and include the impact of system design [106], pH control of the system’s water [113], aeration and filtration technologies [114], acceptable nutrient ranges [115], plant and fish species mating, microbial populations, nitrogen content, feed quantity and type [116], pest management and effective marketing. In a broader sense, aquaponics is an even more complex system involving multiple disciplines, including aquaculture, microbiology, ecology, horticulture, agriculture, chemistry and engineering.

### 4.4. Aquaponic Parameters

In order to optimize the yield of fish and plants alike, the water quality must be adapted to the selected species. The levels of ammonium, nitrate and nitrite are of particular importance here, along with the pH value, the temperature and the oxygen dissolved in the water. High ammonium concentrations are generally toxic for fish. Additionally, the amount of highly toxic ammonia increases automatically with the temperature and the pH value, as these parameters influence the ratio between ammonium and other nitrogen compounds:

NH_4_^+^ ↔ NH_3_ + H^+^. (3)

The pH value is a difficult parameter to adjust, and organisms generally have different demands on it. For the plants, the pH value should not exceed 7.5 in order to optimize the bioavailability of the nutrients. For optimal nitrification, the pH value should be between 6.5 and 8.5. In turn, nitrification lowers the pH by releasing hydrogen ions and utilizing carbon [77].

When nitrate is taken up by the roots of the plants, the pH value will in turn increase because hydroxide ions are being released. Overall, a pH value of about 7 is, thus, also recommended with regard to fish culture. An adequate supply of nitrate is indispensable for plant growth. However, higher concentrations of nitrate in the water lead to the growth inhibition of fish and a simultaneous increase in their stress level [117].

Waste products from aquaculture include dissolved solids, organic and inorganic matter, pathogens from unused food and by-products from fish metabolism such as feces, chemicals or medicines [118]. In particular, the increased amounts of ammonium, nitrate and phosphate in aquacultural wastewater have led to environmental problems. The consequences include the eutrophication of habitats and the direct, harmful effects of excessive ammonium and nitrate on many organisms [119]. The sustainable purification of water through a biofilter and hydroponically cultivated plants thus appears to make sense. Certain plants, namely halophytes, which colonize saline habitats, are particularly suitable for the purification of saline wastewater from aquacultures. The economic feasibility of certain halophytes focusing on their filter performance, including various utilization options, is described in [96].

### 4.5. Aquaponic Organisms

Plants in aquaponic systems must be adapted to the specific composition of the fish effluent. In addition, plant species that have short production cycles with high market value have been preferred. Herbs such as spinach, basil, coriander, chives, purslane, parsley, mint and watercress have already been established in aquaponic systems alongside lettuce. Among others, medicinal and ornamental plants are good candidates for this type of production system. Fish species used in aquaponics include the Spotted Fork Catfish (*Ictalurus punctatus*), the Trout Perch (*Micropterus salmoides*), the Sunfish (*Pomoxis* spp.), the Black Pacu (*Colossoma macropomum*), the Rainbow Trout (*Oncorhynchus mykiss*) and the carp (*Cyprinus carpio*), including the koi, the barramundi (*Lates calcarifer*) and the codfish (*Maccullochella peelii*) named in the Australian Murray Cod. Fish from the African cichlid genus (*Tilapia* spp.) have been most commonly used in aquaponic culture to date [77].

### 4.6. Potential for Phytoremediation

The use of plants for wastewater remediation as a cost-effective and sustainable practice is promising [120]. Phytoremediation is a natural process in which metabolites are degraded in plants and accumulated in the harvested compartments and sometimes even volatilized via plant metabolism. Despite many other plant species, there has been a whole series of studies looking at the potential of watercress for phytoremediation. Organic components such as textile dyes [121], pesticides [122] or pharmaceuticals [123] can be bound in watercress. Inorganic components, especially heavy metals such as arsenic, cadmium, cobalt, copper, chromium, nickel and lead, can also be partially taken up and accumulated in the plants [123,124,125,126,127,128,129,130,131,132,133,134]). For example, watercress tolerates heavy metals at least in moderate amounts and is suitable for the accumulation of cadmium, cobalt, chromium, copper, nickel and lead. Arsenic, on the other hand, interferes with plant growth. In addition, the uptake capacity of watercress for nutrients (nitrogen and phosphorus compounds) has been investigated in a whole series of studies, testing, on the one hand, in hydroponic systems [135,136,137], and on the other hand, in aquaponic systems [138,139]. In summary, the plant tolerates a wide range of nutrient concentrations and has a high nitrogen uptake capacity but also requires relatively high amounts of nitrogen for healthy development.

### 4.7. Preliminary Impact Assessment of Aquaponics

Both aquaponic and hydroponic systems require high energy input, leading to potential environmental burdens. A cradle-to-gate LCA compared the environmental performance, on an economic basis, of aquaponics and hydroponics with identical system designs. For a one-month cultivation period, tilapia and six vegetables produced in the aquaponic system had almost twice the total value of the vegetables from the hydroponic system. Aquaponics produced a 45% lower endpoint environmental impact than hydroponics. Electricity use for heating and lighting and water pumping contributed to the majority of the environmental impacts of both systems, which was followed by the production of fish feed and fertilizers. However, changing the energy source from coal to wind power could make hydroponic systems more environmentally friendly than aquaponic systems [140].

### 4.8. Germany’s Contribution to the Scientific and Economic Development of Aquaponics in the Frame of EU-Funded Projects

INAPRO is an EU-funded project that represents innovative model- and demonstration-based water management for resource-efficient integrated multitrophic systems of vegetable production and aquaculture. Resource-saving solutions are found on a production scale under different geographical and climatic conditions. The aquaponic system was developed by the Leibniz Institute of Freshwater Ecology and Inland Fisheries in the Research Association Berlin e.V. (Forschungsverbund Berlin e.V.; Rudower Chaussee 17; D-12489 Berlin, Germany) and uses ASTAF-PRO technology, which is characterized by an innovative double recirculation system. This is intended to create optimal production conditions in both subsystems for fish and plants. With the help of computer models, sustainable food production was developed that is, according to the researchers, almost emission-free. Cold traps catch the evaporation water in the greenhouse, which is then fed back into the fish section. Even the CO_2_ produced in the fish section is in turn absorbed by the plants to again release O_2_. Aquaponic systems of this kind have already been implemented in Spain, Germany, Belgium and China as part of the project to demonstrate the technical and economic feasibility of the system, and moreover, to open up new market opportunities for manufacturers and operators of aquaponic systems [141].

Another project carried out by IGB Berlin is CITYFOOD, where researchers are developing innovative solutions for integrating multitrophic vegetable production and aquaculture systems (IAAC systems) on a larger scale in urban areas. In this way, the project contributes to meeting the enormous ecological challenges of the future. IAAC systems are resource-efficient and optimize water, energy, wastewater and nutrient dynamics to ensure sustainable urban development. The project uses computer models, real-world experiments in living labs, urban planning aspects and case studies within a multidisciplinary project consortium and disseminates findings to the public in a freely available IAAC knowledge database [142].

Within Germany, aquaponic research has not only been advanced in Berlin. The FischGlasHaus (English: FishGlasHouse), with a total area of 1000 m^2^, was built in 2015 on the campus of the Faculty of Agricultural and Environmental Sciences (AUF), Rostock, and is currently, according to its own information, the most advanced aquaponics experimental facility in Europe. The facility can particularly be characterized by its “multi-unit-system-design” technology. Under ecologically sustainable conditions (no pesticides or antibiotics are used), the influence of different fish species, such as the African predatory catfish (*Clarias gariepinus*) and the Nile tilapia (*Oreochromis niloticus*), on the growth of herbs (basil, mint), cabbages (pak choi) or fruit vegetables (cucumbers) is studied. For plant nutrition, only the wastewater from fish production is used. In addition, the extent to which partial fertilization would be necessary to achieve a marketable plant quality has been examined [143].

European Cooperation in Science and Technology (COST) has been supported by the EU Framework Programme Horizon 2020. The EU project “Aquaponics Hub—Realizing sustainable integrated fish and vegetable production for the EU” (https://www.cost.eu/actions/FA1305/project, accessed on 2 February 2022) has aimed to develop aquaponics in the EU by guiding research through the creation of a networked hub of scientific and industry experts—scientists, engineers, economists, aquaculturists and horticulturists. The project can be divided into three areas. The first area has dealt with the development of aquaponic technologies in cities and urban areas. The second area has been concentrated on development in third world countries to promote food security for local people. The last area has been focused on aquaponic technologies on an industrial scale, exploring competitive systems to produce low-cost, healthy and sustainable food [144].

Berlin has honored its reputation as an innovation hotspot in the private sector as well. In Berlin-Schoeneberg, one of the world’s most modern urban aquaponics facilities, the ECF FARM Berlin, is operated. The aquaponic system produces tilapia and basil and distributes the products regionally to Berlin’s residents. The start-up uses the technologies developed by IGB Berlin as part of the INAPRO project and has equipped the system with two water circuits [145]. The aquaculture cycle for fish production is coupled with the hydroponic cycle for plant production. In this way, two different pH values optimized for the respective cycle can be set. In addition, minerals important for the plants can be added separately as substitute fertilizer without harming the fish. Another big advantage is that the water in this system is used twice, and the fish excretions are used as fertilizer for the plants. In addition to the 1800 m^2^ production area in Berlin, there is an 1100 m^2^ rooftop farm in Bad Ragaz, Czech Republic, for the combined production of trout and lettuce as well as herbs, and another 2400 m^2^ rooftop farm in Brussels, Belgium, where striped bass are produced in the same system as lettuce and herbs [146].

TopFarmers GmbH, also based in Berlin, wants to play a part in the further development of aquaponics, too. With AquaTerraPonik, the waste materials from aquaculture can be utilized by the crops in a closed fish-plant cycle. The uniqueness lies in the fact that special attention is paid to the substrates. They not only serve to hold the plant, but also provide space for many microorganisms and enable an optimal supply of nutrients. With the help of self-developed technologies, such as AgTech, Big Data, IoT, Industry 4.0 and Smart Factory, processes and procedures have been made more transparent and efficient. The networking of sensors and technology plays an important role and provides data for the continuous optimization of the system. As an open platform, the Cloud GrowControl system is self-learning and can be linked with other services [147].

## 5. Vertical Farming

### 5.1. General Aspects of Vertical Farming

Vertical agriculture offers the advantages of a regional supply of fresh food while saving water and avoiding, or at least reducing, the use of pesticides. In doing so, vertical approaches are suitable for mass food production regardless of climate and location. These innovative approaches hold solutions to the intensifying problems of overpopulation and climate change. A driving force for innovation is the new initiative of the Indoor Farming University Network [148], which was founded to support and increase the popularity of the indoor farming industry and connects the worldwide research of vertical approaches.

According to the World Wildlife Foundation’s “2050 Criteria” report, humanity will need to produce more food in the next four decades than we have in the last 8000 years of agriculture combined [149]. Increasing urbanization, in particular, confronts our agriculture with the challenge of supplying growing cities with high-quality food in a sustainable manner. Vertical farming offers a regionally feasible way to meet the increasing food demand of cities. In addition to eliminating pesticides, there are virtually no nutrient emissions and, with an average of only 2–4 L of water per kg of product, huge amounts of water can potentially be saved. Vertical approaches use much less land and potentially fewer resources in the distribution of their products and produce less waste. Alongside this, the controlled environment of an indoor farm not only allows for a significant improvement in quality (taste, flavor, appearance, shelf life, nutritional value, safety) but also the security of continuously high production volumes with consistent quality every day of the year, regardless of weather, climate changes or location [150,151,152,153].

### 5.2. Advantages of Vertical Farming

A major advantage of vertical farming approaches is the controlled indoor condition of the mostly hydroponic or aeroponic cultivation technique. This provides complete control of nutrient supply via optimized fertilization strategies that promote growth, yield and ultimately food quality [154]. This requires that the essential nutrients are available to the plants in the right form, at the right time and in the right quantity. In connection with nutrient deficiencies, morphological growth deficiencies often occur, which limit economic profitability as well as product quality. Growth deficits can be prevented by constant monitoring and, thus, the early detection of nutrient deficits and constant optimization of the fertilization strategy. This in turn requires a basic understanding of the functionality and transport of plant nutrients, which often already enables a visual diagnosis of most nutrient deficiencies [155].

The use of distributed sensors and multivariate mathematical modeling systems is now widespread in modern agriculture. The collection and interpretation of data provide virtually limitless possibilities for optimizing the production apparatus. Vertical agriculture, with its obvious potential for sustainable, water- and land-saving food production, experiences the same optimization potential and can be optimized in exactly the same way as the conventional food industry. The basis of any empirical assessment starts at the level of data and is no less dependent on how you collect it, how you process it and how you respond to it. Very recent developments have been in the field of sensor and processing technology that enable AI-based decision-making, with the goal of consistent and predictable product quality [156].

### 5.3. Disadvantages of Vertical Farming

Indoor farms, often referred to as plant factories with artificial lighting (PFALs), have currently spread due to the postulated advantages of the efficient use of natural resources. However, the major disadvantage of significantly higher energy demand compared to conventional food production systems has been disregarded [157].

Research around the technological features and management protocols of indoor farms has experienced an upsurge in recent years, mainly due to innovations in lighting technologies, cropping systems and environmental control units. In this context, it has been shown that optimal lighting management, especially in terms of spectral composition [158,159], light intensity [160] and photoperiod management [161], can transform overall resource efficiency and environmental performance [162] in controlled crop production systems. A comprehensive analysis of the resource use and environmental impact of such systems is still outstanding.

### 5.4. Preliminary Impact Assessment of Vertical Farming

To systematically assess the environmental advantages and disadvantages of vertical approaches compared to traditional methods of food production, it would be useful to look at an LCA, although only a very limited number of such analyses of vertical or similar systems have been published at present [163].

Conventional arable farming has inherent environmental impacts, such as nitrate leaching, phosphorus losses, nitrous oxide and ammonia emissions, land use and pesticide emissions, to name just a few of them. Negative environmental impacts have reportedly been lower for vertical systems, but this has yet to be verified. Certainly, some disadvantages of vertical approaches, as well as hydroponic systems in general, cannot be dismissed. These include the compulsory use of nutrient solutions, which change in terms of their nutrient composition during cycling and are, at the same time, enriched with various, mostly undesirable organic compounds. For the sustainable and resource-saving use of vertical systems, the question of disposal or reuse of this solution is, thus, unavoidable. In addition, another challenge for vertical systems is presented by their dependence on a massive infrastructure of buildings, racks, containers and lighting. Dealing with this infrastructure considered from manufacturing to disposal is generally resource- and energy-intensive. Another challenge already addressed is electricity consumption. As our power generation becomes more environmentally friendly, the environmental impact of power consumption by agricultural systems is gradually being reduced. Potentially shorter distances from producer to consumer will reduce transportation processes overall, which would need to be evaluated in detail, but can generally be seen as a benefit. However, the LCA evaluation would also need to consider whether the incorporation of vertical farms in cities has an impact on other transportation activities, including personnel transportation. Additionally, the LCA should also take into account the land consumption associated with vertical systems to make the argument for lower land requirements comprehensible in reality. In this context, the goal is to obtain future electricity from renewable sources, which in turn could massively increase land requirements, considering the large areas for solar panels or wind turbines. So far, the argument for lower water consumption can mainly be used to justify the use in arid regions, but less so in areas where natural irrigation prevails thanks to sufficient water reserves. It is often mentioned that no pesticides are used in vertical or general hydroponic systems. This conceals the fact that the use of toxic biocides to disinfect vertical systems is sometimes well-considered. All in all, the potential of vertical systems to increase the sustainability of our food production has yet to be critically and objectively evaluated, which is not to say that it is not there. In particular, the handling of the nutrient solution, including its disposal or reuse, is a key challenge for vertical as well as hydroponic systems [164].

## 6. Rough Comparison of Soilless Systems

Aeroponics is, by definition, a subset of hydroponics, with the difference that no growing medium is used in any case. It is a method of cultivation in which the plants are anchored in holes in polystyrene sheets and their roots are suspended in the air below the sheet. Aeroponic cultivation is usually practiced in sheltered structures and is suitable for low leafy vegetables such as lettuce, spinach, etc. [86].

By contrast, aquaponics is a sustainable food production system that combines the hydroponic culture of plants with aquaculture in a closed water cycle [94]. The aquaponic system relies on the combination of animals and plants to maintain a stable and natural aquatic environment. The natural functioning in aquaponics means that the elements tend to balance each other out. In addition, hydroponics is set up in a clean, man-made environment, while aquaponics is a replica of a natural ecosystem’s functioning, favoring organic cycles. The fish waste forms the organic nutrient source for the growing plants, and the plants provide a natural filter, especially for the toxic nitrogen residues from the fish waste. Microbes (nitrifying bacteria) usually grow in a separate biofilter and convert the ammonium from the fish waste first into nitrite and afterwards into nitrate, which then can be reused by the plants [165].

At first glance, the three farming systems of hydroponics, aeroponics and aquaponics have some aspects in common, as they do not use soil as a substrate for growing crops. Similarly, they are supposed to deliver sustainable and profitable food production. The difficulty in comparing hydroponics and aquaponics lies in the different prerequisites of the systems. The construction of an aquaponic system itself is already more labor- and cost-intensive, as stages for the filtration and treatment of the fish wastewater have to be set up, as well as an aquarium as a habitat for the fish themselves. Nevertheless, there are some significant differences where aquaponics ensures advantages over hydroponics, such as the higher cost of fertilizer to provide plant nutrients in the hydroponic system. In the aquaponic system, the nutrients from the fish excrements are reused for conversion by microorganisms and subsequently to build up new plant material. Several studies have concluded that aquaponics farming generally shows faster and more efficient results in terms of plant growth compared to hydroponics. In terms of nutrient solution control, it is not necessary to upload the nutrient solution in the aquaponic system, while uploading and disposing of the nutrient solution in the hydroponic system may well become necessary, as salts and other by-products accumulate and become toxic to the plants. In an established aquaponic system, pH and nitrogen levels only need to be monitored once a week and nitrate levels just once a month as a result of the natural balancing of these components. Aquaponics can, thus, show greater water-use efficiency. In addition, in an aquaponic system, the electrical conductivity does not need to be checked once a day as it does in a commercial hydroponic system, which reduces labor input, too [165].

Although aeroponics and hydroponics are designed to be similar in their use of nutrient-rich water, they are distinctly different. Hydroponics often uses media other than soil to retain and distribute nutrient-rich water to nourish the plants. In contrast, aeroponics uses a misting system to deliver the nutrients. Aeroponics promises greater efficiency in vertical growing arrangements and efficient use of space. Both the hydroponic and aeroponic systems allow for flexibility and control of quality, health and quantity. At the same time, hydroponics can use only up to 10% of water resources compared to conventional methods. Aeroponics has no higher water-saving potential as it does not use a nutrient medium and instead sprays a nutrient-rich solution onto the root system to maximize nutrient uptake. While an aeroponic system requires constant monitoring, hydroponic systems can be relatively user-friendly. However, both systems are probably more efficient in terms of their use of water and space and their yield performance than soil-based agriculture, and both have almost the same ability to flexibly control irrigation and nutrient applications [28].

Aeroponic systems allow for a reduction of water consumption by up to 98%, fertilizer use by up to 60% and pesticide use by almost 100%. At the same time, crop yields can be maximized. Plants grown aeroponically can, in some cases, absorb more nutrients, which makes the plants more nutritionally valuable. Another advantage of aeroponics is that the plants can be transferred with less effort, as they do not suffer from transplant shock. A disadvantage in the hydroponic system is the transfer of nutrient solution between plants, which makes it possible for diseases to transfer rapidly between plants via the water [166].

## 7. Conclusions

In recent years, soilless cultivation has become increasingly important as a promising strategy for growing a variety of crops. This approach provides the opportunity to grow short-lived crops, such as vegetables, throughout the whole year with comparatively few land and labor requirements. Especially in regions with limited water or soil resources, hydroponic cultivation techniques can open up new approaches to food production. To support this development, cost-effective hydroponic technologies that save labor as well as operating costs through increased automation need to reach the market. On the other hand, hurdles such as the risk of the rapid spread of diseases within the closed systems, as well as contradictions such as the need for fossil energy resources have to be overcome. As long as sustainability is limited by the need for fossil resources, large building structures, technical equipment, any disinfectants and waste materials, the use of hydroponic techniques should always be critically considered in terms of environmental balance and long-term consequences for both the planet’s health and ours. When evaluating current research results, there is a lack of comparable sustainability studies. Efforts are needed to obtain clear data, especially regarding the environmental impact of high energy costs, and to find alternatives to fossil energy sources. In order to be able to more accurately assess the future opportunities of soilless techniques, it is necessary to know the advantages and disadvantages of the individual systems, substrates and organisms and to understand their applicability. Not all systems are equally efficient, nor can they be applied in all areas and locations. Similarly, not all plant species are equally suitable for cultivation in soilless systems. Economic efficiencies cannot be neglected here. The substrates used in some cases also have a separate impact on the environment, which must be critically included in the overall consideration. So, in addition to the lighting and nutrient supply, the numerous systems, applications, substrates and organisms and their economic viability and sustainability have to be considered in order to get an understanding of whether and when it is worthwhile to use soilless techniques.

## Appendix A

**Table A1:** Liquid or solution culture techniques further subdivided into circulating (closed) and non-circulating (open) techniques. GM, growth medium.

Technique	Description	Advantages	Disadvantages
Circulating methods (closed systems)	Nutrient solution pumped through plant root system, collecting and reusing excess solution.		
NFT [23,31,43,110]	Plants in channels with nutrient solution flowing past roots driven by a slight gradient. Often in substrate-filled net pots.	- low initial costs - reusability of nutrient solution - low probability of blockages	- control of nutrient concentrations and pH required - difficult for species with short roots - power/pump failures
DFT [23,31,43,167]	Growing directly in nutrient solution. Often in substrate-filled net pots.	- efficient water and nutrient use - water-loving and fast-growing plants - can be organic	- take care regarding sufficient oxygenation - risk of diseases, etc.
Ebb and flow system [23,31,168,169]	Plants placed directly in growing trays often filled with medium. Nutrient solution periodically floods planting chamber and returns to reservoir.	- water-loving plants - energy-efficient - easily scalable	- high demand for reservoir capacities and nutrient medium - risk of anoxia - power/pump failures
Drip system [23,31,33,43,170]	Substrate in which roots are supplied with nutrient solution via drip emitter, often periodically.	- simple installation - efficient water and nutrient use and easy control	- drip lines/emitters susceptible to blockages - control of nutrient concentrations and pH required - power/pump failures
Wick system [23,43,171]	Capillary action feeds plants via synthetic fibers such as nylon. Often with absorbent medium.	- suitable for indoor, small or single plants such as herbs and spices - passive; no electricity needed	- requires a lot of water
Aeroponic [28,166,172]	Roots hang in air surrounded by sufficient oxygen and wetted with aerosol of nutrient solution distributed by atomizers.	- excellent aeration - reusability of nutrient solution - absence of GM - water-saving - low risk of diseases - high productivity - vertical usability	- maintenance effort - atomizers may clog - cleaning of root chambers - high initial costs - power/pump failures - control of nutrient concentrations and pH required
Aquaponic [28,77,95]	Combining hydroponic culture of plants with aquaculture of fish in closed water cycle and nitrifying bacteria.	- efficient water and nutrient use - no fertilizers required	- maintenance effort - risk of algae growth - risk of diseases, etc. - power/pump failures - sufficient oxygenation
Non-circulating methods	Nutrient solution is replaced when nutrient concentration decreases or pH and EC change.		
Root dipping technique [173]	Pots closely spaced submerged in nutrient solution.	- inexpensive - little maintenance - passive; no electricity needed - good aeration	- less efficient water and nutrient use - risk of diseases, etc.
Floating technique [27]	Plants in small pots are fixed to Styrofoam sheet (or light plate) and float on nutrient solution.	- inexpensive - little maintenance - passive; no electricity needed	- less efficient water and nutrient use - risk of diseases, etc. - artificial aeration
Capillary action technique [174]	Plant pots with holes at bottom and inert medium placed in shallow containers. Nutrient solution reaches medium by capillary action.	- suitable for ornamental, flower and indoor plants	- aeration depending on medium

**Table A2:** Solid media culture techniques or aggregate systems.

Technique	Description	Advantages	Disadvantages
Hanging bag technique [173]	Approximately 1 m long polyethylene bags filled with sterilized coconut fibers are sealed at the bottom and hung up (“verti-grow” technique). Plants in net pots pressed into holes in the sides of hanging bags. Nutrient solution distributed evenly from the top by micro-sprinkler. The nutrient solution drips down and moistens coconut fibers and plant roots.	- chance to reuse solution through nutrient solution-collecting channel - suitable for lettuce, leafy vegetables, strawberry and small flower plants	- risk of algae or mold growth - power/pump failures
Grow bag technique [175]	Polythene bags about 1 m long filled with sterilized coconut dust are placed horizontally in rows on the ground. Small holes are made at the top of the bags and 2–3 plants in net pots per bag are pressed into them. Slits on each side of the bags are provided for drainage, and fertilization is done with a black capillary tube leading from the main feed line to each plant.	- chance to reuse solution through nutrient solution-collecting channel	- risk of algae or mold growth - power/pump failures
Trench or trough technique [176]	Plants are grown in narrow trenches or in above-ground stone troughs. Inner linings of trenches are covered by thick polythene sheets. Size and shape of trench are constructed according to cropping nature. All required nutrients with water are circulated through the dripping system with a pump.	- suitable for lettuce, coriander, spinach, etc. - chance to reuse solution if nutrient solution-collecting channel is used	- tall-growing vine plants (cucumber, tomato, etc.) need additional support to carry the weight of their fruits - risk of algae or mold growth - power/pump failures
Pot technique [27]	Pot technique is similar to trench or trough culture, but growing media is filled in clay or plastic pots.	- chance to reuse solution if nutrient solution-collecting channel is used - greater controllability by singling out	- tall-growing plants need additional support - risk of algae or mold growth - power/pump failures

**Table A3:** List of different plant species that can be cultivated hydroponically. Summarized partly after Hayden [44], Resh [83], Singh and Singh [32], Sardare and Adame [86], Mohammed [43] and Sharma et al. [33].

Type of Crop	Plant Species	Expected Yield	References
Cereals	*Avena sativa* (Oat)	Comparable or higher	Al-Karaki and Al-Momani [177], Fazaeli et al. [178], Singh and Singh [32]
	*Glycine max* (Soybean)	Higher and greater quality	Palermo et al. [179], Singh and Singh [32]
	*Oryza sativa* (Rice)	Cultivar-dependent, higher	Vargas-Rodríguez [180], Singh and Singh [32], Irfan et al. [181]
	*Pisum sativum* (Peas)	Comparable or higher	Singh and Singh [32], Jada, M. A. S. al [182]
	*Triticum aestivum* (Wheat)	Higher	Gros et al. [183], Du Toit and Labuschagne [184], Singh and Singh [32]
	*Zea mays* (Maize)	Comparable or higher	Vargas-Rodríguez [180], Rivera et al. [185], Singh and Singh [32], Bhattacharya [186]
Fruits	*Cucumis melo* (Melons)	Higher and greater quality	Guler et al. [187], Fukuda and Anami [188], Yam et al. [189]
	*Fragaria ananassa* (Strawberry)	Lower	Sarooshi and Cresswell [190], Albaho et al. [191], Treftz and Omaye [192]
	*Rubus idaeus* (Raspberry)	Higher	Treftz and Omaye [193]
	*Vaccinium corymbosum* (Blueberry)	Higher	Nascimento et al. [194]
	*Vitis vinifera* (Grapes)	Comparable	Nemati et al. [55]
Vegetables	*Allium cepa* (Onion)	Comparable	Pascual et al. [195]
	*Allium fistulosum* (Scallion)	NA	
	*Allium schoenoprasum* (Chive)	Comparable	Resh [83]
	*Apium graveolens* (Celery)	Comparable	Pascale et al. [196]
	*Asparagus officinalis* (Asparagus)	Lower	Poll et al. [197]
	*Beta vulgaris* (Beet)	Higher	Singh and Singh [32]
	*Brassica oleracea var.* botrytis (Cauliflower)	Higher	Singh and Singh [32]
	*Brassica oleracea var.* capitata (Cabbage)	Higher	Singh and Singh [32]
	*Brassica oleracea* var. *sabellica* (Kale)	Higher	Chandra et al. [198]
	*Capsicum annum* (Bell pepper)	Lower or Higher	Albaho et al. [191], Chandra et al. [198]
	*Capsicum frutescens* (Chili)	Higher	Alimuddin et al. [199]
	*Cucumis sativus* (Cucumbers)	Comparable or higher	Gros et al. [183], Singh and Singh [32], Chandra et al. [198]
	*Cucurbita pepo* (Zucchini)	Higher	Chandra et al. [198]
	*Lycopersicon esculentum* (Tomato)	Higher	Singh and Singh [32], Chandra et al. [198]
	*Psophocarpus tetragonolobus* (Winged bean)	Comparable or lower	Chow and Price [200]
	*Raphanus sativus* (Radish)	Comparable	Gros et al. [183]
	*Solanum melongena* (Eggplant)	Higher	Costa et al. [201]
	*Solanum tuberosum* (Potato)	Comparable or higher	Ritter et al. [202], Singh and Singh [32]
Leafy vegetables	*Atriplex* spp. (Saltbush)	Comparable	Sharma et al. [33]
	*Beta vulgaris* subsp. *vulgaris* (Swiss chard)	Higher	Maboko and Plooy [203], Chandra et al. [198]
	*Ipomoea aquatica* (Kang Kong)	Semi-aquatic plant	Xiang et al. [204]
	*Lactuca sativa* (Lettuce)	Higher	Singh and Singh [32], Barbosa et al. [205], Touliatos et al. [38]
	*Spinacia oleracea* (Spinach)	Higher	Ranawade et al. [40]
Condiments	*Anethum graveolens* (Dill)	Comparable	Resh [83]
	*Anthriscus cerefolium* (Chervil)	Comparable	Resh [83]
	*Artemisia dracunculus* (Tarragon)	Comparable	Resh [83]
	*Barbarea verna* (Upland cress)	Comparable	Resh [83]
	*Brassica integrifolia* (Mustard)	Higher	Padmathilake et al. [206]
	*Foeniculum vulgare* (Fennel)	Comparable	Resh [83]
	*Mentha x piperita* (Peppermint)	Higher	Daryadar [207], Mairapetyan et al. [208]
	*Mentha spicata* (Mint)	Higher and greater quality	Hayden [44], Padmathilake et al. [206], Vimolmangkang et al. [209], Surendran et al. [210]
	*Nasturtium officinale* (Watercress)	Comparable	Resh [83]
	*Ocimum* spp. (Basil)	Comparable or higher and greater quality	Sgherri et al. [211], Resh [83], Chandra et al. [198], Mairapetyan et al. [208]
	*Origanum majorana* (Marjoram)	Comparable	Resh [83]
	*Origanum vulgare* (Oregano)	Comparable	Resh [83]
	*Petroselinum crispum* (Parsley)	Higher	Chandra et al. [198]
	*Salvia officinalis* (Sage)	Comparable	Resh [83]
	*Thymus vulgaris* (Thyme)	Comparable	Resh [83]
	*Trachyspermum roxburghianum* (Asamodagam)	Higher	Padmathilake et al. [206]
	*Trigonella foenum-graecum* (Methi)	Higher	Gurdas et al. [212]
Flower/ornamental crops	*Chrysanthemum indicum* (Chrysanthemum)	Higher	Wilson and Finlay [213]
	*Dianthus caryophyllus* (Carnations)	Higher	Hanan and Holley [214]
	*Rosa berberifolia* (Roses)	Comparable	Das et al. [215]
	*Tagetes patula* (Marigold)	Comparable	Sarmah and Bora [216]
Medical crops	*Aloe vera* (Indian Aloe)	Higher	Bhattacharya [186]
	*Anemopsis californica* (Yerba mansa)	Lower	Hayden [44]
	*Solenostemon scutellarioides* (Coleus)	NA	
	*Urtica dioica* (Nettle)	Comparable	Hayden [44]
Fodder crops	*Axonopus compressus* (Carpet grass)	NA	
	*Cynodon dactylon* (Bermuda grass)	NA	
	*Hordeum vulgare* (Barley)	Comparable	Asadullah et al. [37], Al-Karaki and Al-Hashimi [217]
	*Medicago sativa* (Alfalfa)	Comparable	Al-Karaki and Al-Hashimi [217]
	*Sorghum bicolor* (Sorghum)	Comparable or higher	Vargas-Rodríguez [180], Bhattacharya [186]

**Table A4:** Comparison of different substrates/GM modified after Savvas and Gruda [31].

Material	Origin	Advantages	Disadvantages
Sand	Natural with particles of 0.05–2.0 mm	Relatively inexpensive, good drainage ability	Low nutrient- and water-holding capacity, high volume weight, low total pore space
Rockwool	Melted silicates at 1500–2000 °C	Light volume weight, high total pore space, ease of handling, totally inert, nutrition can be carefully controlled	Disposal problems, energy consumed during manufacture
Vermiculite	Mg, Al and Fe silicate sieved and heated to 1000 °C	Light volume weight, high nutrient-holding ability, good water-holding ability, good pH buffering capacity, good aeration due to high pore space	Compacts when too wet, energy-consuming product, expensive
Perlite	Siliceous volcanic mineral sieved and heated to 1000 °C	Light volume weight, sterile, neutral in pH (6.5–7.5), no decay, sufficient total pore space	Low nutrient capacity, energy-consuming product, expensive
Pumice	Light silicate mineral of volcanic material	Relatively light volume weight, good total pore space, cheap and long-lasting, environmentally friendly	High transport costs, pH may be high
Peat	Natural anaerobically processed plant residues	Physical stability, good air and water-holding capacity due to high total pore space, low microbial activity, light volume weight, low and easily adjustable pH, low nutrient content	Finite resource, environmental concerns (CO_2_ release), increasing cost due to energy crisis, may be strongly acidic, shrinking may lead to substrate hydro-repellence
Coconut coir	By-product of fiber coconut processing	Physical stability, light weight, good air content due to high total pore space and high water-holding capacity, subacid-neutral pH (5–6.8)	May contain high salt levels, energy consumption during transport
Bark (well-aged)	By-product or waste of wood manufacture	Good air content and water-holding capacity, good total pore space, sub-acid-neutral pH (5–7), sufficient volume weight, long-lasting	High variability, need time to reduce C:N ratio and terpenes concentrations, increasing cost since used as an alternative to fuel and in landscaping
Green compost	Composted plant residues	Good source of potassium and micronutrients, suppression of diseases, good moisture-holding capacity, urban waste reduction	Non-homogeneous, high volume weight, may contain excess salt, need time to be composted, becomes easily waterlogged
Biochar and hydrochar	Solid material derived from biomass pyrolysis or biomass hydrolysis	Production energy-neutral, helps with carbon sequestration, biologically very stable, wet material can be used for hydrochar; hydrochar has low electric conductivity	Properties vary dependent on feedstock (biochar), high production costs, biochar often has high pH, can be dusty
